# Ocular Signs Related to Overweight and Arterial Hypertension in Children: A Systematic Review

**DOI:** 10.2174/1874364101711010273

**Published:** 2017-08-31

**Authors:** Daniela S. Schuh, Ângela B. Piccoli, Raquel L. Paiani, Cristiane R Maciel, Lucia C Pellanda, Manuel AP Vilela

**Affiliations:** Instituto de Cardiologia/Fundacao Universitária de Cardiologia, Porto Alegre, Brazil; Universidade Federal de Ciências da Saúde de Porto Alegre, Porto Alegre, Brazil

**Keywords:** Child obesity, Systemic arterial hypertension, Retinopathy, Retinal vessels, Eyes abnormalities

## Abstract

**Background:**

The ocular effects of obesity and hypertension need to be established and can be used as prognostic markers.

**Objective:**

To estimate the prevalence of ophthalmological alterations in children and adolescents who are overweight and/or have SAH.

**Methods:**

The database for this study included all observational studies (CS, cohort, case-control and “baseline” description of randomized clinical trials) with children and/or adolescents who were overweight, obese or had SAH and that measured ophthalmological alterations.

**Results:**

Comparative studies with healthy children demonstrated positive association between body adiposity with retinal venular dilation, and SAH with retinal arteriolar narrowing. Different retinal fundus cameras and computer-assisted programs to evaluate the retinal vessels, variations in the methods of analysis, adjustments, populations, were the main arguments against formal meta-analysis. The heterogeneity was too high (I^2^ >90%, in fixed or randomized effects), and the lack of linearity, normal distribution and homoscedasticity did not recommend meta-regression.

**Conclusion:**

Obesity and SAH show associations with ophthalmological alterations, especially with retinal vessel diameter. Lack of standardization does not allow a quantitative evaluation.

## 1 INTRODUCTION

Child obesity has been recognized as a global public health problem which can negatively affect almost all the body’s organs and cause relevant disorders such as asthma, early onset of puberty, glomerulopathy, misaligned lower limbs, hypertension, cardiovascular disease, white brain matter damage and mortality [1-6]. Worldwide, there are 1.1 billion obese and overweight people and 10%-25% of children are estimated to be affected by this situation [7-9]. Recent estimates calculate that there are between 42.4 and 51.8 million children and adolescents aged 0-18 in this context in Latin America [8].

With regard to children in particular, the mechanisms underlying the association between obesity and SAH and microvascular disease, however, have yet to be enlightened. Assessment of retinal circulation has been proposed as a way of estimating systemic microcirculation and detecting the first signs of systemic cardiovascular and cerebrovascular disease in clinical practice [10-12].

The morphology and flow in the retinal vessels (caliber, tortuosity, branching patterns, fractal dimensions, flow) are markers frequently analyzed. It is almost consensus that in adults the narrower the retinal arteriolar vessel, the greater the possibility of association with SAH [13], cerebrovascular accidents [11, 12, 14] and coronary artery disease [15, 16]; whilst the wider the retinal vein diameter, the greater the associations with systemic inflammation and obesity [17, 18]. Cross-sectional(CS) population-based studies have shown that increased body mass indices (BMI) are associated with retinal arteriolar narrowing and increased retinal venular diameter among adults [19-21]. These findings are in agreement with data from studies with healthy child populations, indicating a possible effect of obesity and/or SAH on early alterations to microvascular structure in childhood [22-25].

Despite the evidence, the results regarding independent association between obesity (with or without SAH) and alterations in retinal vessels caliber (RVC) in children and adolescents are heterogeneous and the mechanisms are still unclear [26]. The only review on this topic includes perinatal risk factors (low weight, gestational age), environmental factors (physical activity, diet, family hypertension), systemic factors (hypertension, obesity) and diseases associated with future cardiovascular changes. However, it limited searches to a few databases, restricted languages, included only articles accessible to the authors' library, and did not make a qualitative risk analysis of the studies [27].

Studies involving adult populations do not have full control over confounding factors inherent to this age group relating to arterial pressure, diabetes, smoking, use of medication and it is therefore of interest to know the results of studies conducted with healthy children and youth [22]. The body mass index (BMI) in children is considered *normal* between percentile 3 and 85; *low weight* below percentile 3; *overweight* between percentile 85 and 97 and *obesity* when above percentile 97, as established by the World Health Organization in 2007 for children and adolescents (10-19 years-old). These take into consideration adolescent growth spurt [26].

This study therefore seeks to assess, by means of a systematic review of observational studies, the presence of ophthalmological alterations in children and adolescents who are overweight or have SAH.

## 2 METHODS

### 2.1 Protocol and Registration

This systematic review was conducted in accordance with Cochrane [28], PRISMA [29] (“Preferred Reporting Items for Systematic Reviews and Meta-Analysis”), and MOOSE [30] (“Meta-analysis of Observational Studies in Epidemiology”) criteria, and has been registered on the PROSPERO database [31] (“International Prospective Register of Systematic Reviews”).

### 2.2 Eligibility Criteria

All articles published up until May 2017 held on the MEDLINE (“Medical Literature Analysis and Retrieval System Online”) via PubMed; Embase; Scopus; Web of Science; BVS databases were considered, with no restriction as to language. Manual searches in periodicals, references used in included articles and citation analysis were also taken into consideration.

The database for this study included all observational studies (CS, cohort, case-control and “baseline” description of randomized clinical trials) with children and/or adolescents, aged 6 to 18, with cardiovascular risk factors (overweight, obesity or hypertension) which had ophthalmological abnormalities or alterations as their outcome.

The initial search used the MeSH terms “Child”, “Adolescent”, “Overweight”, “Obesity”, “Pediatric Obesity”, “Hypertension”, “Visual Acuity”, “Blindness”, “Visual Perception”, “Vision, Ocular”, “Retina”, “Retinal Diseases”, “Retinal Vessels”, “Refractive Errors”, “Eye Injuries”, “Amblyopia”, “Vision, Binocular”, “Glaucoma”, “Cataract”, “Optic Nerve Injuries”, “Hypertensive Retinopathy”, “Ocular Hypertension”, “Optic Disk”, as well as related publication titles. The full search strategy used for the PubMed database is shown in Appendix 1.

### 2.3 Study Selection

**Fig. (1):**
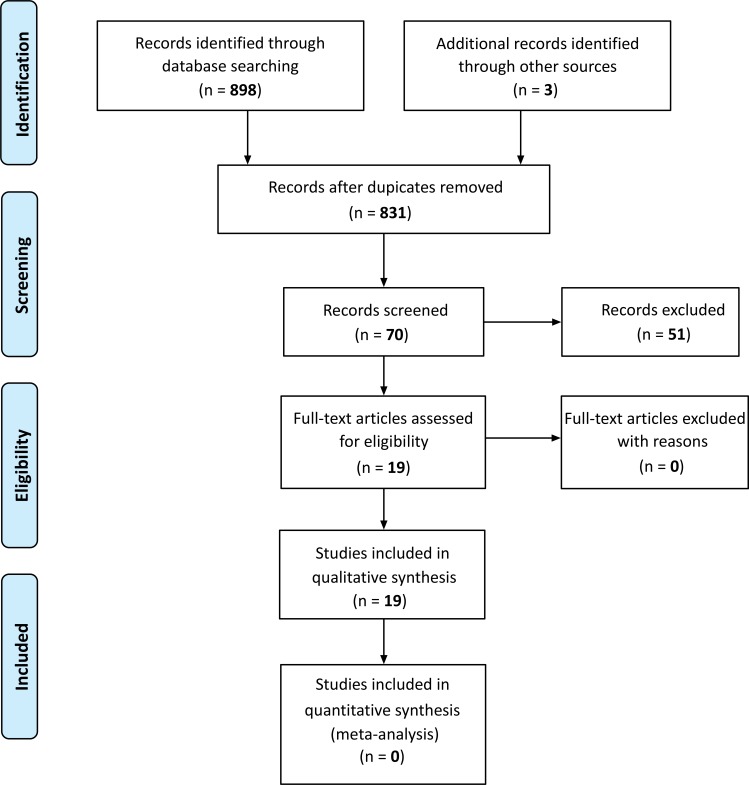
Study flowchart.

The articles selected from the databases were divided into two batches under the responsibility of two reviewers each, so that there were four different reviewers overall. Article titles and abstracts were assessed independently by each reviewer. When abstracts did not provide sufficient information as to the eligibility criteria, the entire text was assessed. The reviewers assessed the full text of the articles independently from each other and determined study eligibility. Disagreements were solved by consensus and if they persisted the opinion of a fifth reviewer specialized in the theme in question was sought (Fig. **1**).

### 2.4 Study Quality

Risk of bias was assessed in each study according to the Newcastle-Ottawa Scales [32] and the scales proposed by Loney **et al*.* [33] The following parameters were considered in the case of cohort studies: sample representativeness, eligibility criteria, ascertainment of exposure, demonstration that the outcome of interest was not present at the start of the study, design or analysis comparability, results evaluation, and sufficient follow-up time for the results to occur. The following parameters were considered in the case of CS studies: adequacy of study design and sampling method, sample size, appropriateness of sampling frame, objective, suitable and standard criteria used for the measurement of health outcome; unbiased assessment; response rates, description of losses, appropriate expression of prevalence or incidence estimates detailed by subgroups, given with confidence intervals; study subject and results. The items considered with regard to case-control studies were case representativeness, control definition and selection, case and control comparison on the basis of design, case ascertainment, use of the same method to ascertain cases and controls, same rate for both groups, and description of losses.

### 2.5 Data Extraction

Four reviewers worked independently; two reviewers worked on the analysis of 50% of the articles and the other two reviewers worked on the other 50%. Disagreements were settled by a fifth reviewer. Overall qualitative or quantitative characteristics of the studies were gathered, such as: nature of the study, population of children/youths aged 6-18, outcome relating to ophthalmological alterations based on assessment of BMI, weight and blood pressure.

### 2.6 Data Analysis

It was not possible to perform a meta-analysis owing to the discrepancy in the methods used to measure the outcomes in the studies and the discrepancies between the data reported in the articles obtained using these methods. Different retinal fundus cameras and computer-assisted programs to evaluate the retinal vessels, variations in the methods of analysis, adjustments, populations, were the main arguments against formal meta-analysis. The heterogeneity was too high (I^2^ >90%, in fixed or randomized effects), and the lack of linearity, normal distribution and homoscedasticity did not recommend meta-regression.

## 3 RESULTS

Fig. (**1**) shows that 901 studies were identified, 19 of which were included in this analysis. Three studies assessed both SAH and overweight [34, 36, 37]. Further five studies assessed SAH [10, 38-41] and the remaining eleven studies assessed the effects of overweight [6, 22, 24, 25, 35, 42-47], as described in Tables (**1** and **2**). The methodological quality of the studies is described in (Table **3**).

### 3.1 SAH and Ocular Manifestations

With regard to intraocular pressure (IOP), two series had contrary results. Yang **et al*.* [34] assessed 1565 children aged 11.9 years on average and found association between the highest quartiles of diastolic pressure and IOP, whereby IOP increased 0.5 mmHg for every 10 mmHg increase in arterial pressure. Akinci **et al*.* [35] did not find association between IOP and SAH. The differences between these findings lie in their different methods and samples: the tonometry methods were not similar and the populations came from diverse ethnic groups.

Axial length (AL) and spherical equivalent (SE) did not show association with SAH and variations were attributed to the ethnic characteristics of the samples. Mitchell *et al*. [10] studied a cohort of two populations (1952 individuals) and in the Sydney sample AL was 22.6±0.7 mm and SE was +1.3±0.9 dioptres (D), whereas in the Singapore sample AL was 24.2±1.0 mm and SE was -2.25±2.01 D. Kurniawan *et al*. [40] studied a sample of 1174 children in Singapore and found 23.27±0.93 mm AL and -0.33±1.67 D SE.

Reduction in retinal artery caliber was the finding most expressively associated with SAH. This was demonstrated in all the studies. In the six quantitative studies, it appeared as a significant fundoscopic marker [40-42]. In the qualitative study conducted by Daniels **et al*.* [38], it was described in 41% of the children in the last quartile of SAH. For every 10 mmHg increase in arterial pressure, Gopinath **et al*.* [39] found a reduction of 2.08μ in Sydney children and 1.43μ in Singapore children, whilst Mitchell *et al*. [10] found a reduction of 1.64-2.11μ. Retinal venous caliber showed no relationship with SAH.

### 3.2 Obesity and Ocular Manifestations

Four series assessed IOP in obese children [34, 35, 44, 46]. Yang **et al*.* [34] (1565 children, non-contact tonometry) and Albuquerque **et al*.* [44] (96 individuals; applanation tonometry) did not find association. Akinci **et al*.* [35] detected a 10% increase in IOP among obese children (applanation tonometry); when analyzing 5919 individuals with an average age of 10.0±3.3 years (non-contact tonometry), Jiang **et al*.* [46] found association with IOP in obese females (p<0.001).

Saw *et al*. [42] found association between obesity and hypermetropia in boys; Kurniawan **et al*.* [47] detected in the entire cohort -2.23±1.45 D SE, although unrelated to obesity. An isolated finding in the series studied by Akinci **et al*.* [35] was the detection of exophthalmia in 18% of the children using the Hertel instrument. The majority of the studies found significant association between obesity and dilated retinal veins. Kurniawan *et al*. [47] found that for every 1 kg/m^2^ increase in BMI, retinal venular caliber increased by 0.59μ. Cheung **et al*.* [22] found a relationship between 3.1 kg/m^2^ BMI and 2.55μ dilation in the same caliber. Diverging results were found with regard to retinal arteriolar caliber. Gopinath **et al*.* [25], Yau **et al*.* [6], Xiao *et al*. [26] and Gishti *et al*. [45] described retinal arteriolar narrowing in these cases; Cheung **et al*.* [22], Kurniawan **et al*.* [47] and Li **et al*.* [43] did not find association between retinal artery diameter and obesity.

## 4 DISCUSSION

According to the findings of our systematic review, children with SAH and/or obesity may have retinal repercussions similar but not identical to those observed in adults, in particular arteriolar narrowing in hypertensive children and venular dilation in children with high BMI. This is due to several factors, including the status of the retinal vessels at the time of the onset of SAH, the duration, severity and response to its management as well as prevalent comorbidities (diabetes, hypercholesterolaemia) [10, 13, 18, 19].

This evidence comes from both direct measurement and also from analyses based on the principle of the fractal dimension, whereby rarefaction of the retinal vessels can be seen [38-42]. The following mechanisms have been proposed regarding venous dilation among the obese: increased blood volume and increased leptin levels (the latter stimulating nitric acid synthesis), and increased pre-inflammatory factors (TNFα) or inflammatory factors [38].

As such, these potential markers can be a basis for supporting diagnosis and control of these situations in childhood. Control of these factors could reduce the consequences for the target organs [48]. These vascular conditions leave traces of their presence from the onset and the marker can be used for following up on suspected cases. The difficulty lies in the routine inclusion of expensive retinal images and digital measuring systems for these vessels, considering that qualitative analysis restricts long-term follow-up of these findings to subjectivity. Moreover, as yet there is no standardized and universal acceptance or use of these quantitative estimation devices.

It must be taken into consideration that measurements made by computerized equipment are automatic estimates derived from non-uniform criteria or based on images subject to discrepancies. Moreover, the vascular zones analyzed are not always equivalent. In cases of axial myopia, or perinatal morbidities the retinal vessels tend to be narrower and these aspects may confound the results.

Based on the studies selected here, unanimity is lacking with regard to association between IOP and SAH and/or obesity [34, 35, 42, 44, 46]. Differing populations, different measurement methods (including body position), as well as the lack of more relevant data, such as corneal thickness, analysis of the optic disc and visual fields, prevent undeniable association from being established. The mechanisms suggested include (1) excessive intraorbital fat and blood hyperviscosity compromising venous drainage, (2) increased aqueous humour filtration owing to greater circulatory flow in the ciliary arteries, and (3) body position during tonometry, assuming that obese patients binding forward and often straining may cause alterations in measurements. It is appropriate to highlight that small differences in eye pressure measurements are not always clinically relevant [35].

The findings regarding refractive status and axial length were not different to those found in normal weight children [10, 34, 40, 42, 46]. Xiao and colleagues [26] found that microvascular alterations are only present in children over 12 years old, implying that obesity may be associated with microvasculature only in children near to puberty.

In adults and also similarly in children above 6 years old, the relationship between SAH and obesity, especially with regard to retinal vessels, has been demonstrated in a large number of publications. Arteriolar narrowing associated with SAH and venular dilation associated with obesity are markers of these situations. A recent systematic review and meta-analysis of 10229 adult cases concluded that in adults retinal arteriolar narrowing and retinal venular dilation are associated independently and significantly with higher risk of SAH, especially in people aged over 60. The study estimated that for every 1.12 mmHg increase in arterial pressure there was arteriolar reduction of 20μ. This data is relevant both for case prognosis and control [49].

According to the META-EYE Study Group [7] regarding a meta-analysis involving 44000 individuals, retinal arteriolar caliber reduces and venular caliber increases in obese adults when compared to those with normal weight, regardless of cardiovascular risk factors. In this case, an increase of 1 kg/m^2^ is associated with a difference of 0.07μ [95%CI:-0.08-0.06] in arteriolar caliber and 0.22μ (95%CI:021-0.23] in venous caliber. Given that these amounts correspond to a less than 1% in relation to normal calibers, their clinical significance is not clear [7]. In children these proportions regarding artery calibers in SAH and vein calibers in cases with high BMI appear to be considerably similar [22]. Even in those aged under six years old it was demonstrated that BMI and systolic arterial pressure have inverse linear associations with retinal artery caliber, and BMI has a positive linear association with vein caliber. For every kg /m^2^ increase in BMI, there was a 1.06 µm reduction in retinal artery caliber (*p* = 0.01) and 1.12 µm widening of vein caliber (*p* = 0.02). The findings were also similar in those whose BMI was ≥ 95%. And for every 10 mm Hg increase in systolic pressure a 1.70 µm reduction was found in retinal artery caliber (*p* = *0.02*) [50]. So subtle differences are difficult to perceive in ophthalmoscopy, and depends on specific equipment.

The strength of this systematic review lies in the bringing together of specific studies with children, with no restriction as to language, to form a significant sample pool. There are many limitations: (1) different populations, non-standardized and diverse methods used to assess variables, resulting in possible overestimation or underestimation, (2) the absence of prospective studies, preventing an appreciation of the causal link and the evolution of the findings over time, (3) different or imprecise criteria principally in relation to defining obesity. It was also found that the use of BMI does not accurately reflect the form of adipose tissue distribution, nor does it distinguish fat deposition from muscle hypertrophy [38].

## CONCLUSION

In conclusion, children with SAH and/or obesity suffer alterations in the dimensions of their retinal blood vessels. With the aid of digital photography and special computerized equipment, it is possible to measure these alterations, but these resources have yet to be standardized. Evolving studies will be essential to determine whether the findings described can or cannot be transformed into markers.

## Figures and Tables

**Table:** 

Search	Add to Builder	Query	Items Found	Time
#4	Add	Search **(#1 AND #2 AND #3)**	901	13:40:17
#3	Add	Search **(“Child”[MESH] OR “Children”[Title/Abstract] OR “Adolescent”[MESH] OR “Adolescents”[Title/Abstract] OR “Adolescence”[Title/Abstract] OR “Teens”[Title/Abstract] OR “Teen”[Title/Abstract] OR “Teenagers”[Title/Abstract] OR “Teenager”[Title/Abstract] OR “Youth”[Title/Abstract] OR “Youths”[Title/Abstract] OR “Adolescents, Female”[Title/Abstract] OR “Adolescent, Female”[Title/Abstract] OR “Female Adolescent”[Title/Abstract] OR “Female Adolescents”[Title/Abstract] OR “Adolescents, Male”[Title/Abstract] OR “Adolescent, Male”[Title/Abstract] OR “Male Adolescent”[Title/Abstract] OR “Male Adolescents”[Title/Abstract])**	2671967	13:39:16
#2	Add	Search **(“Overweight”[Mesh] OR “Obesity”[Mesh] OR “Pediatric Obesity”[Mesh] OR “Obesity, Pediatric”[Title/Abstract] OR ”Adolescent Obesity”[Title/Abstract] OR “Obesity, Adolescent”[Title/Abstract] OR “Obesity in Adolescence”[Title/Abstract] OR “Childhood Obesity”[Title/Abstract] OR “Obesity, Childhood”[Title/Abstract] OR “Childhood Onset Obesity”[Title/Abstract] OR “Obesity, Childhood Onset”[Title/Abstract] OR “Onset Obesity, Childhood”[Title/Abstract] OR “Obesity in Childhood”[Title/Abstract] OR “Child Obesity”[Title/Abstract] OR “Obesity, Child”[Title/Abstract] OR “Dyslipidemias”[Mesh] OR “Dyslipidemia”[Title/Abstract] OR “Dyslipoproteinemias”[Title/Abstract] OR “Dyslipoproteinemia”[Title/Abstract] OR “Hypertension”[Mesh] OR “Blood Pressure, High”[Title/Abstract] OR “Blood Pressures, High”[Title/Abstract] OR “High Blood Pressure”[Title/Abstract] OR “High Blood Pressures”[Title/Abstract])**	419714	13:38:31
#1	Add	Search **(“Visual Acuity”[MESH] OR “Acuities, Visual”[Title/Abstract] OR “Acuity, Visual”[Title/Abstract] OR “Visual Acuities”[Title/Abstract] OR “Amaurosis Fugax”[MESH] OR “Monocular Blindness, Transient”[Title/Abstract] OR “Blindness, Transient Monocular”[Title/Abstract] OR “Transient Monocular Blindness”[Title/Abstract] OR “Blindness, Monocular, Transient”[Title/Abstract] OR “Blindness”[MESH] OR “Blindness, Acquired”[Title/Abstract] OR “Acquired Blindness”[Title/Abstract] OR “Blindness, Monocular”[Title/Abstract] OR “Monocular Blindness”[Title/Abstract] OR “Blindness, Hysterical”[Title/Abstract] OR “Hysterical Blindness”[Title/Abstract] OR “Blindness, Transient”[Title/Abstract] OR “Transient Blindness”[Title/Abstract] OR “Blindness, Legal”[Title/Abstract] OR “Legal Blindness”[Title/Abstract] OR “Amaurosis”[Title/Abstract] OR “Amauroses”[Title/Abstract] OR “Blindness, Complete”[Title/Abstract] OR “Complete Blindness”[Title/Abstract] OR “Visual Perception”[MESH] OR “Perception, Visual”[Title/Abstract] OR “Perceptions, Visual”[Title/Abstract] OR “Visual Perceptions”[Title/Abstract] OR “Vision, Ocular”[MESH] OR “Ocular Vision”[Title/Abstract] OR “Vision”[Title/Abstract] OR “Light Signal Transduction, Visual”[Title/Abstract] OR “Visual Phototransduction”[Title/Abstract] OR “Phototransduction, Visual”[Title/Abstract] OR “Visual Transduction”[Title/Abstract] OR “Transduction, Visual”[Title/Abstract] OR “Visual Light Signal Transduction”[Title/Abstract] OR “Retina”[MESH] OR “Retinas”[Title/Abstract] OR “Retinal Diseases”[MESH] OR “Disease, Retinal”[Title/Abstract] OR “Diseases, Retinal”[Title/Abstract] OR “Retinal Disease”[Title/Abstract] OR “Retinal Vessels”[MESH] OR “Retinal Vessel”[Title/Abstract] OR “Vessel, Retinal”[Title/Abstract] OR “Vessels, Retinal”[Title/Abstract] OR “ Retinal Blood Vessels”[Title/Abstract] OR “Blood Vessel, Retinal”[Title/Abstract] OR “Blood Vessels, Retinal”[Title/Abstract] OR “Retinal Blood Vessel”[Title/Abstract] OR “Vessel, Retinal Blood”[Title/Abstract] OR “Vessels, Retinal Blood”[Title/Abstract] OR “Pecten Oculi”[Title/Abstract] OR “Retinopathy of Prematurity”[MESH] OR “Prematurity Retinopathies”[Title/Abstract] OR “Prematurity Retinopathy”[Title/Abstract] OR “Retrolental Fibroplasia”[Title/Abstract] OR “Fibroplasia, Retrolental”[Title/Abstract] OR “Fibroplasias, Retrolental”[Title/Abstract] OR “Retrolental Fibroplasias”[Title/Abstract] OR “Refractive Errors”[MESH] OR “Error, Refractive”[Title/Abstract] OR “Errors, Refractive”[Title/Abstract] OR “Refractive Error”[Title/Abstract] OR “Refractive Disorders”[Title/Abstract] OR “Disorder, Refractive”[Title/Abstract] OR “Disorders, Refractive”[Title/Abstract] OR “Refractive Disorder”[Title/Abstract] OR “Ametropia”[Title/Abstract] OR “Ametropias”[Title/Abstract] OR “Eye Injuries”[MESH] OR “Eye Injury”[Title/Abstract] OR “Injury, Eye”[Title/Abstract] OR “Injuries, Eye”[Title/Abstract] OR "Amblyopia"[Mesh] OR “Amblyopias”[Title/Abstract] OR “Lazy Eye”[Title/Abstract] OR “Eye, Lazy”[Title/Abstract] OR “Eyes, Lazy”[Title/Abstract] OR “Lazy Eyes”[Title/Abstract] OR “Anisometropic Amblyopia”[Title/Abstract] OR “Amblyopia, Anisometropic”[Title/Abstract] OR “Amblyopias, Anisometropic”[Title/Abstract] OR “Anisometropic Amblyopias”[Title/Abstract] OR “Amblyopia, Developmental”[Title/Abstract] OR “Amblyopias, Developmental”[Title/Abstract] OR “Developmental Amblyopia”[Title/Abstract] OR “Developmental Amblyopias”[Title/Abstract] OR “Amblyopia, Suppression”[Title/Abstract] OR “Amblyopias, Suppression”[Title/Abstract] OR “Suppression Amblyopia”[Title/Abstract] OR “Suppression Amblyopias”[Title/Abstract] OR “Stimulus Deprivation-Induced Amblyopia”[Title/Abstract] OR “Stimulus Deprivation Induced Amblyopia”[Title/Abstract] OR “Amblyopia, Stimulus Deprivation-Induced”[Title/Abstract] OR “Amblyopia, Stimulus Deprivation Induced”[Title/Abstract] OR “Amblyopias, Stimulus Deprivation-Induced”[Title/Abstract] OR “Deprivation-Induced Amblyopia, Stimulus”[Title/Abstract] OR “Deprivation-Induced Amblyopias, Stimulus”[Title/Abstract] OR “Stimulus Deprivation-Induced Amblyopias”[Title/Abstract] OR “Vision, Binocular”[MESH] OR “Binocular Vision”[Title/Abstract] OR “Glaucoma”[MESH] OR “Glaucomas”[Title/Abstract] OR “Strabismus”[MESH] OR “Squint”[Title/Abstract] OR “Phorias”[Title/Abstract] OR “Phoria”[Title/Abstract] OR “Strabismus, Noncomitant”[Title/Abstract] OR “Noncomitant Strabismus”[Title/Abstract] OR “Mechanical Strabismus”[Title/Abstract] OR “Strabismus, Mechanical”[Title/Abstract] OR “Strabismus, Comitant”[Title/Abstract] OR “Comitant Strabismus”[Title/Abstract] OR “Convergent Comitant Strabismus”[Title/Abstract] OR “Comitant Strabismus, Convergent”[Title/Abstract] OR “Strabismus, Convergent Comitant”[Title/Abstract] OR “Hypertropia”[Title/Abstract] OR “Hypertropias”[Title/Abstract] OR “Cataract”[MESH] OR “Cataracts”[Title/Abstract] OR “Lens Opacities”[Title/Abstract] OR “Lens Opacity”[Title/Abstract] OR “Opacities, Lens”[Title/Abstract] OR “Opacity, Lens”[Title/Abstract] OR “Cataract, Membranous”[Title/Abstract] OR “Cataracts, Membranous”[Title/Abstract] OR “Membranous Cataract”[Title/Abstract] OR “Membranous Cataracts”[Title/Abstract] OR “Pseudoaphakia”[Title/Abstract] OR “Pseudoaphakias”[Title/Abstract] OR “Optic Nerve Injuries” [MESH] OR “Injuries, Optic Nerve”[Title/Abstract] OR “Injury, Optic Nerve”[Title/Abstract] OR “Nerve Injuries, Optic”[Title/Abstract] OR “Nerve Injury, Optic”[Title/Abstract] OR “Optic Nerve Injury”[Title/Abstract] OR “Optic Nerve Trauma”[Title/Abstract] OR “Nerve Trauma, Optic”[Title/Abstract] OR “Nerve Traumas, Optic”[Title/Abstract] OR “Optic Nerve Traumas”[Title/Abstract] OR “Trauma, Optic Nerve”[Title/Abstract] OR “Traumas, Optic Nerve”[Title/Abstract] OR “Trauma, Second Cranial Nerve”[Title/Abstract] OR “Second Cranial Nerve Injuries”[Title/Abstract] OR “Second Cranial Nerve Trauma”[Title/Abstract] OR “Cranial Nerve II Injuries”[Title/Abstract] OR “Optic Neuropathy, Traumatic”[Title/Abstract] OR “Neuropathies, Traumatic Optic”[Title/Abstract] OR “Neuropathy, Traumatic Optic”[Title/Abstract] OR “Optic Neuropathies, Traumatic”[Title/Abstract] OR “Traumatic Optic Neuropathies”[Title/Abstract] OR “Traumatic Optic Neuropathy”[Title/Abstract] OR “Optic Nerve Contusion”[Title/Abstract] OR “Contusion, Optic Nerve”[Title/Abstract] OR “Contusions, Optic Nerve”[Title/Abstract] OR “Nerve Contusion, Optic”[Title/Abstract] OR “Nerve Contusions, Optic”[Title/Abstract] OR “Optic Nerve Contusions”[Title/Abstract] OR “Optic Nerve Transection”[Title/Abstract] OR “Nerve Transection, Optic” OR “Nerve Transections, Optic”[Title/Abstract] OR “Optic Nerve Transections”[Title/Abstract] OR “Transection, Optic Nerve”[Title/Abstract] OR “Transections, Optic Nerve”[Title/Abstract] OR “Optic Nerve Avulsion”[Title/Abstract] OR “Avulsion, Optic Nerve”[Title/Abstract] OR “Avulsions, Optic Nerve”[Title/Abstract] OR “Nerve Avulsion, Optic”[Title/Abstract] OR “Nerve Avulsions, Optic”[Title/Abstract] OR “Optic Nerve Avulsions”[Title/Abstract] OR “Hypertensive Retinopathy”[MESH] OR “Hypertensive Retinopathies”[Title/Abstract] OR “Retinopathies, Hypertensive”[Title/Abstract] OR “Retinopathy, Hypertensive”[Title/Abstract] OR “Diabetic Retinopathy”[MESH] OR “Diabetic Retinopathies”[Title/Abstract] OR “Retinopathies, Diabetic”[Title/Abstract] OR “Retinopathy, Diabetic”[Title/Abstract] OR “Ocular Hypertension”[MESH] OR “Hypertension, Ocular”[Title/Abstract] OR “Hypertensions, Ocular”[Title/Abstract] OR “Ocular Hypertensions”[Title/Abstract] OR “Glaucoma, Suspect”[Title/Abstract] OR “Glaucomas, Suspect”[Title/Abstract] OR “Suspect Glaucoma”[Title/Abstract] OR “Suspect Glaucomas”[Title/Abstract] OR “Optic Disk”[MESH] OR “Disk, Optic”[Title/Abstract] OR “Disks, Optic”[Title/Abstract] OR “Optic Disks”[Title/Abstract] OR “Optic Papilla”[Title/Abstract] OR “Optic Papillas”[Title/Abstract] OR “Papilla, Optic”[Title/Abstract] OR “Papillas, Optic”[Title/Abstract] OR “Optic Nerve Head”[Title/Abstract] OR “Head, Optic Nerve”[Title/Abstract] OR “Heads, Optic Nerve”[Title/Abstract] OR “Nerve Head, Optic”[Title/Abstract] OR “Nerve Heads, Optic”[Title/Abstract] OR “Optic Nerve Heads”[Title/Abstract] OR “Blind Spot”[Title/Abstract] OR “Blind Spots”[Title/Abstract] OR “Spot, Blind”[Title/Abstract] OR “Spots, Blind”[Title/Abstract])**	507949	13:36:45

**Table 1:** Characteristics of the Studies assessing Hypertension, or Hypertension and Obesity as a Risk Factor

Author (year)	Design	Sample (n)	Age - years (m±sd)	Male Sex n (%)	Main characteristics relating to hypertension	Outcome(s)	Result(s)
Daniels *et al.* (1991) [38]	CS	97	14.7 ±3.5	73 (75.2)	AP (last quartile)	Qualitative analysis of vessel caliber during retinography	41% narrowing, 14% tortuous, 8% intertwined a/v
Mitchell *et al.* (2007) [10]	CS	1952	Sidney 6.3±0.4 Singapore 7.6±0.6	Sidney 992 (50.8) Singapore 1109 (56.8)	AP (mean and quartiles)	Refraction + Biometry + Quantitative analysis of vessel caliber during retinography	Spherical Equivalent (D): +0.4 to -4.25 Axial Length (mm): 21.9 to 25.2 RAC reduced in the highest quartile RVC not associated
Gopinath et al. (2010) [39]	CS	2272	12.7±0.4	1154 (50.8)	AP (mean and quartiles)	Quantitative analysis of vessel caliber during retinography	CAV reduced RVC not associated
Hanssen *et al.* (2012) [36]	CS	578	11.1 ±0.6	329 (56.9)	AP BMI> 90th percentile	Quantitative analysis of vessel caliber during retinography	RAC reduced RVC not associated
Kurniawan, E.D. *et al.* (2012) [40]	CS	1174	11.9 ± 1.0	576 (49.1)	AP, SP, DP	Refraction + Biometry + Quantitative analysis of caliber and circulation pattern in vessels during retinography	Spherical Equivalent (dioptres): -0.33 (±1.67) not related Axial Length (mm): 23.27 (±0.9) not related Retinal Fractal Dimension reduced RAC reduced RVC not related
Murgan *et al.* (2013) [41]	CS	121	16.2±1.5	68 (56.2)	AP >90th percentile 90th percentile, but below 95th percentile, or if arterial pressure exceeded 120/80 mmHg	Quantitative analysis of vessel caliber during retinography	RAC reduced in SAH RVC not related Relação caliber a/v: not related
Yang et al. (2014) [34]	CS	1565	11.9 ±3.5	801 (51.2)	AP last quartile and BMI	Refraction + Biometry + Intraocular Pressure (IOP): (non-contact)	SE (D): -1.58±2.0 (no relationship) AL(mm): 17.1±3.6 (no relationship) IOP associated with diastolic pressure; not related to BMI
Imhof *et al.* (2016) [37]	CS	391	7.3 +- 0.4	191 (48.8)	AP>95th percentile BMI (WHO)	Quantitative analysis of vessel caliber during retinography	SAH:RAC reduced RVC not related; Obesity: RAC/RVC not related after adjustment for blood pressure

AL= axial length of the globe of the eye, AP=arterial pressure, BMI= body mass index, D=dioptre, DP= diastolic pressure, Intertwined a/v = artery/vein intertwined, IOP= intraocular pressure, Narrowing = retinal arteriolar narrowing, RAC= retinal arteriolar caliber, RVC= retinal venular caliber, SE= spherical equivalent, SP= systolic pressure, sd= standard deviation, CS= cross-sectional.

**Table 2:** Characteristics of the Studies assessing Obesity as a Risk Factor

Author (year)	Design	Sample (n)	Age (m±sd)	Male Sex n (%)	Main characteristics relating to obesity	Outcome(s)	Result(s)
Saw et al. (2002) [42]	CS	1449	8 (±1)	746 (51.5)	BMI (quartiles)	Refraction	Weight related to hypermetropy boys Height related to myopia
Akinci *et al.* (2007) [35]	Case-control	72 (BMI >95) 72 (BMI <95)	Group I Boys 13.7 (2.6) Girls 14.6(1.8) Group C Boys 13.6(2.7) Girls 14.5(1.9) >0.05	49 (68)	BMI >95^th^ percentile	Exophtham (Hertel) IOP (applanation)	18% obese with exophthalmia IOP not associated with blood pressure levels 10% obese with increased IOP
Cheung *et al.* (2007) [22]	CS	768	7.9±0.8	403 (52.5)	BMI (quartiles)	Quantitative analysis of vessel caliber during retinography	RAC: not associated RVC: increased (every 3.1 kg/m^2^ BMI increases vein 2.55μ)
Gopinath *et al.* (2011) [24]	CS	2353	12.7 ± 0.4	1188 (50.5)	BMI (IOTF)	Quantitative analysis of vessel caliber during retinography	RAC: decreased RVC: increased
Li *et al.* (2011) [43]	CS	136	11.19 ± 2.5	69 (50.1)	BMI > 85% percentile Triceps skin fold	Quantitative analysis of vessel caliber during retinography	RAC: not associated RVC: increased
Albuquerque et al (2013) [44]	CS	96	11 ± 2.8	46 (48)	BMI ≥ 95% percentile	IOP (applanation)	IOP not associated
Jiang et al. (2014) [46]	CS	5919	10.0±3.3	3118 (52.7)	BMI	IOP (non-contact)	IOP associate with BMI in boys
Kurniawan *et al.* (2014) [47]	Cohort	421	11.9 ± 0.8	207 (49.2)	BMI (WHO)	Refraction + Quantitative analysis of vessel caliber during retinography	SE (D): -2.23±1.4 RAC: no relationship RVC: increased
Yau et al. (2014) [6]	CS	39 cases 51 controls	17.8 ±1.6	15 (38.4)	IR (QUICKI)	Quantitative analysis of vessel caliber during retinography	RAC: decreased RVC: increased
Gishti *et al.* (2015) [45]	CS	4145	6.0 +- 0.3	2072 (50)	BMI ≥ 95% percentile	Quantitative analysis of vessel caliber during retinography	RAC decreased RVC not related
Xiao et al. (2015) [25]	CS	731	Subgroup 1: 7-11 y Girls 9.4±1.2 Boys 9.62±1.36 Subgroup 2: 12-19 a Girls 14.5±2 Boys 14.3±2.4	339 (46.3)	BMI	Quantitative analysis of vessel caliber during retinography	RAC and RVC: between 12-19 years narrowing related to adiposity

AP= arterial pressure, BMI= body mass index, IOP= intraocular pressure, IOTF= International Obesity Task Force, IR (QUICKI) = index of resistance to insulin and method, RAC= retinal arteriolar caliber, RVC= retinal venular caliber, WHO= World Health Organization, sd= standard deviation, CS= cross-sectional, Exophtham = exophthalmometry

**Table 3:** Summary of Study Quality (with all scales used)

Reference		Are the study design and sampling method appropria-te for the research question?		Is the sampling frame appropri-ate?		Is the sample size adequate?		Are objective, suitable and standard criteria used for measure-ment of the health outcome?		Is the health outcome measured in an unbiased fashion?		Is the response rate adequate? Are the refusers described?			Are the estimates of prevalence or incidence given with confidence intervals and in detail by subgroup, if appropriate?		Are the study subjects and the setting described in detail and similar to those of interest to you?		Score	
(6)		1		1		ND		1		1		1			1		1		7	
(10)		1		1		ND		1		1		1			1		1		7	
(22)		1		1		ND		1		1		1			1		1		7	
(24)		1		1		ND		1		1		1			1		1		7	
(25)		1		1		ND		1		1		1			1		1		7	
(34)		1		1		ND		1		1		1			1		1		7	
(36)		1		1		ND		1		1		1			1		1		7	
(37)		1		1		ND		1		1		1			1		1		7	
(38)		-		-		ND		1		1		1			-		1		5	
(39)		1		1		ND		1		1		1			1		1		7	
(40)		1		1		ND		1		1		1			1		1		7	
(41)		1		1		ND		1		1		1			1		1		7	
(42)		1		1		ND		1		1		1			1		1		7	
(43)		1		1		ND		1		1		1			1		1		7	
(44)		1		1		ND		1		1		1			1		1		7	
(45)		1		1		ND		1		1		1			1		1		7	
(46)		1		1		ND		1		1		1			1		1		7	
Reference		Representativeness of the exposed cohort		Selection of the non exposed cohort		Ascertainment of exposure		Demonstration that outcome of interest was not present at start of study		Comparability of cohorts on the basis of the design or analysis		Assessment of outcome			Was follow-up long enough for outcomes to occur		Adequacy of follow-up of cohorts		Newcastle - Ottawa Quality Assessment Scale Cohort Studies	
(47)		*		*		*		*		**		*					*		8	
Reference	Is the case definition adequate?		Representativeness of the cases		Selection of the controls		Definition of controls		Comparability of cases and controls on the basis of the design or analysis		Ascertainment of exposure		Same method of ascertainment for cases and controls	Same rate for both groups		Non respondents described		Rate different and no designation	Total
(35)	*				*		*		*		*		*	*					7*

ND = not determined
